# Knee Cartilage Thickness Differs Alongside Ages: A 3-T Magnetic Resonance Research Upon 2,481 Subjects via Deep Learning

**DOI:** 10.3389/fmed.2020.600049

**Published:** 2021-02-09

**Authors:** Liping Si, Kai Xuan, Jingyu Zhong, Jiayu Huo, Yue Xing, Jia Geng, Yangfan Hu, Huan Zhang, Qian Wang, Weiwu Yao

**Affiliations:** ^1^Department of Imaging, Tongren Hospital, Shanghai Jiao Tong University School of Medicine, Shanghai, China; ^2^Institute for Medical Imaging Technology, School of Biomedical Engineering, Shanghai Jiao Tong University, Shanghai, China; ^3^Department of Radiology, The First Affiliated Hospital, College of Medicine, Zhejiang University, Hangzhou, China; ^4^Department of Radiology, Shanghai Jiao Tong University Affiliated Sixth People's Hospital, Shanghai, China; ^5^Department of Radiology, Ruijin Hospital, Shanghai Jiao Tong University School of Medicine, Shanghai, China

**Keywords:** knee, cartilage, deep learning, MR, thickness

## Abstract

**Background:** It was difficult to distinguish the cartilage thinning of an entire knee joint and to track the evolution of cartilage morphology alongside ages in the general population, which was of great significance for studying osteoarthritis until big imaging data and artificial intelligence are fused. The purposes of our study are (1) to explore the cartilage thickness in anatomical regions of the knee joint among a large collection of healthy knees, and (2) to investigate the relationship between the thinning pattern of the cartilages and the increasing ages.

**Methods:** In this retrospective study, 2,481 healthy knees (subjects ranging from 15 to 64 years old, mean age: 35 ± 10 years) were recruited. With magnetic resonance images of knees acquired on a 3-T superconducting scanner, we automatically and precisely segmented the cartilage via deep learning and calculated the cartilage thickness in 14 anatomical regions. The thickness readings were compared using ANOVA by considering the factors of age, sex, and side. We further tracked the relationship between the thinning pattern of the cartilage thickness and the increasing ages by regression analysis.

**Results:** The cartilage thickness was always thicker in the femur than corresponding regions in the tibia (*p* < 0.05). Regression analysis suggested cartilage thinning alongside ages in all regions (*p* < 0.05) except for medial and lateral anterior tibia in both females and males (*p* > 0.05). The thinning speed of men was faster than women in medial anterior and lateral anterior femur, yet slower in the medial patella (*p* < 0.05).

**Conclusion:** We established the calculation method of cartilage thickness using big data and deep learning. We demonstrated that cartilage thickness differed across individual regions in the knee joint. Cartilage thinning alongside ages was identified, and the thinning pattern was consistent in the tibia while inconsistent in patellar and femoral between sexes. These findings provide a potential reference to detect cartilage anomaly.

## Introduction

Osteoarthritis (OA) is a chronic, disabling joint disease with a total incidence of 15% in populations (1, 2). OA causes prevalent damages to many parts of the knee joint, including articular cartilage and subchondral bone, which then seriously impair the quality of life of middle-aged and older people (3, 4). The osteoarthritic chondrocytes have undergone comprehensive degeneration and structural disorders (5). Pieces of evidence suggested that early degeneration in cartilage also impaired subchondral bone and incurred bone remodeling (6, 7). The current therapeutic approach for OA was largely palliative (8). Due to the unknown pathogenesis, there was no effective treatment other than pain relief (9) and joint replacement in the advanced stage of the disease (10).

Therefore, it is necessary to systematically analyze structural changes of knee cartilage by magnetic resonance (MR) imaging to better understand the development of OA and to focus on early detection and disease prevention. Previous studies reported cartilage/meniscus segmentation and quantification (11), evaluation of knee cartilage lesions (12), and measurement of femoral cartilage thickness (13). Several researchers inferred cartilage thickness using the joint space of plain films and small-scale femoral cartilage thickness (14). However, few efforts in the literature were devoted to systematically analyzing the cartilages of the whole joint and provided comprehensive baseline data on cartilage thickness of the individual regions-of-interest (ROIs) in the knee joint, which now became technically feasible by combining big imaging data and artificial intelligence, with the ubiquitous application of deep learning in medical imaging in the past years (15, 16).

Concerning the necessity to examine the critical role of articular cartilage in the whole knee joint, we conducted a comprehensive and in-depth big data study to track the thickness changes of knee cartilage in this work. Specifically, we quantified the cartilage morphology by automatic image segmentation powered by deep learning and standardize the knee cartilage thickness among a large Chinese population of healthy knees. We aim to compare the cartilage thickness among different age groups and associate the thickness readings with 14 individual ROIs in the knee joint according to the whole-organ magnetic resonance imaging score (WORMS) (17). In this way, our research could provide new insights into the cartilage thickness thinning pattern of normal knees and could potentially act as a reference to detect the anomaly and to track disease progress.

## Materials and Methods

### Participants

**Figure 1:**
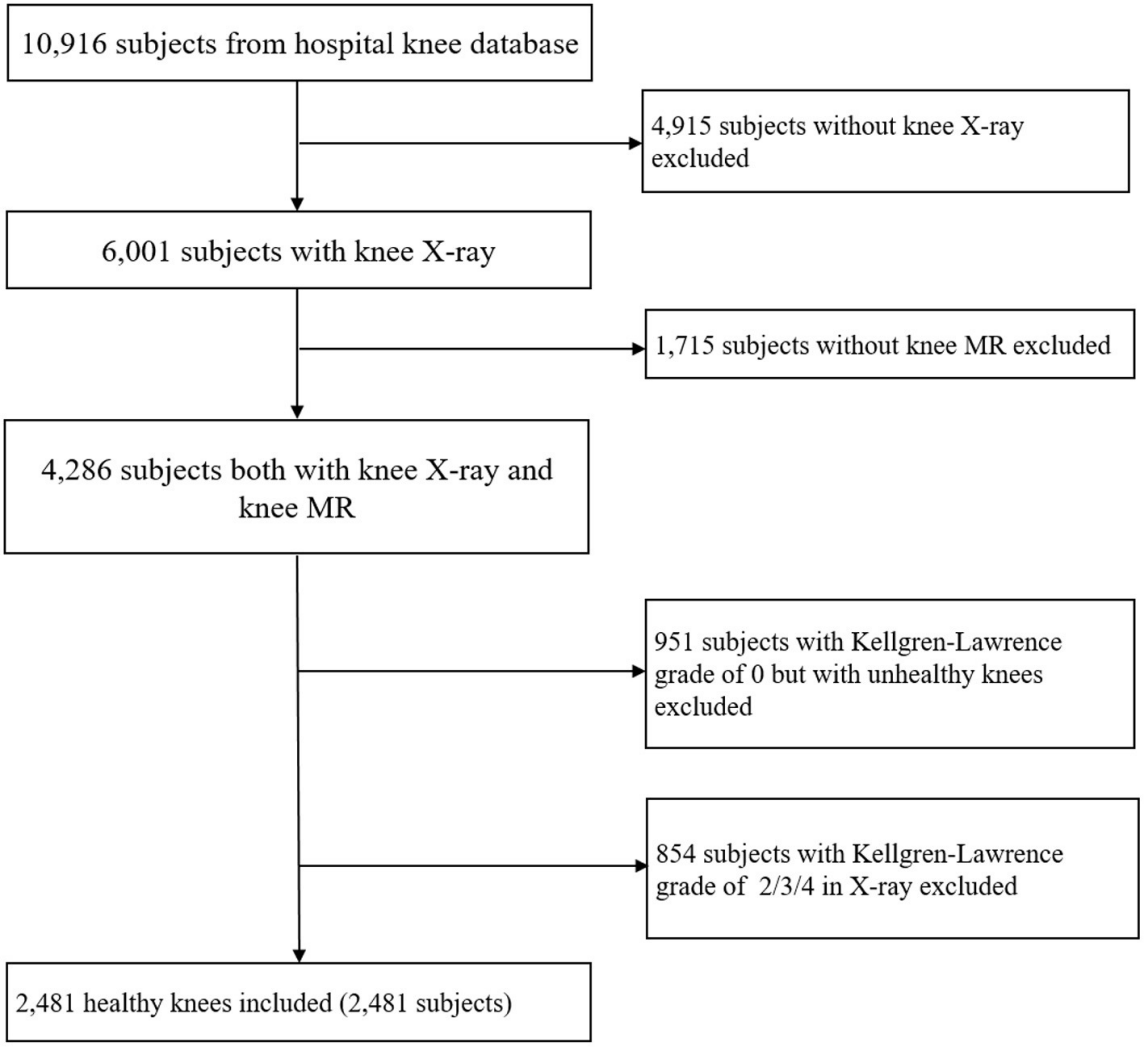
Flowchart of data selection and subjects used in study.

This research project was approved by the institutional review board, Tongren Hospital, Shanghai Jiao Tong University School of Medicine. Subjects were retrospectively selected from the hospital database with both plain films and MR images from January 2018 to March 2019. Figure 1 provided our flowchart to select the subjects. Inclusion criteria were knees without OA, i.e., having a Kellgren-Lawrence grade of 0 (18, 19), which was reviewed by two practicing board-certified musculoskeletal radiologists at a large academic practice (years in practice 10–20 years) independently. Finally, 2,481 subjects were included, and the ages of the subjects ranged from 15 to 64 years old, with mean age: 35 ± 10 years; male: 1,355 subjects (35 ± 10 years); female: 1,126 subjects (36±11 years); left knee: 1,228 subjects (36±11 years), right knee: 1,253 subjects (35 ± 10 years). The informed consent requirement was waived, as this study was a retrospective review of radiologic images without identification and health information of patients.

Images of the knees were acquired on a 3-T superconducting MR scanner (Achieva 3.0TX; Philips Healthcare, Best, Netherlands) with an eight-channel knee coil (Philips Healthcare). The knee flexion angle was kept naturally bent, and a dedicated holder was used to reduce motion artifacts at the time of acquisition. The MR protocol included four sequences (Table 1): (1) sagittal T1W sequence: repetition time/echo time (TR/TE) = 638/20 ms, field of view (FOV) = 160 mm, matrix = 260 × 208, section thickness = 3 mm, bandwidth = 289 KHz; (2) sagittal T2W fat-suppressed sequence: TR/TE = 3,004/62 ms, FOV = 100 mm, matrix = 292 × 188, section thickness = 3 mm, bandwidth = 218 KHz; (3) coronal proton density-weighted fat-suppressed sequence: TR/TE = 2,137/25 ms, FOV = 160 mm, matrix = 324 × 168, section thickness = 3 mm, bandwidth = 218 KHz; (4) transversal proton density-weighted fat-suppressed sequence: TR/TE = 2,351/25 ms, FOV = 160 mm, matrix = 324 × 168, section thickness = 3 mm, bandwidth = 218 KHz.

**Table 1:** Parameters for imaging sequences in MRI examination.

**Parameters**	**Sagittal T1WI**	**Sagittal T2W FS**	**Coronal PDW FS**	**Transversal PDW FS**
TR (ms)	638	3004	2137	2351
TE (ms)	20	62	25	25
Flip angle (degrees)	90	90	90	90
FOV (cm)	160	100	160	160
Matrix size	260 × 208	292 × 188	324 × 168	324 × 168
Section thickness (mm)	3	3	3	3
Bandwidth (KHz)	289.6	217.9	218.0	218.0
File type	DICOM	DICOM	DICOM	DICOM

*PD, proton density; DICOM, digital information and communications in medicine; TIWI, T1-weighted imaging; T2W FS, T2-weighted fat-suppressed; PDW FS, PD-weighted fat-suppressed; TR, repetition time; TE, echo time; FOV, field of view*.

### Structural Regions

**Figure 2:**
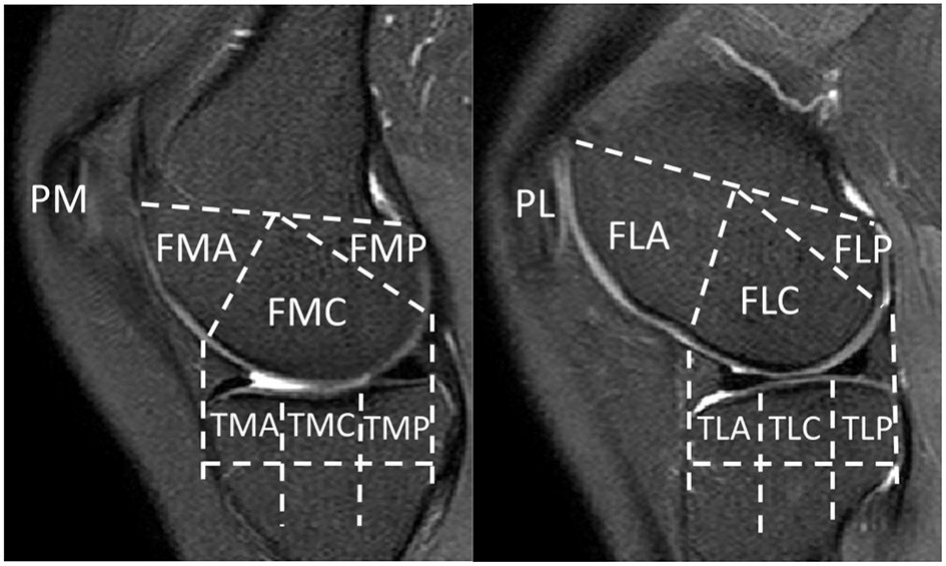
Knee MR images were divided into 14 structural regions-of-interest (ROIs). FMA, medial anterior femur; FMC, medial central femur; FMP, medial posterior femur; FLA, lateral anterior femur; FLC, lateral central femur; FLP, lateral posterior femur; TMA, medial anterior tibia; TMC, medial central tibia; TMP, medial posterior tibia; TLA, lateral anterior tibia; TLC, lateral central tibia; TLP, lateral posterior tibia; PM, medial patella; PL, lateral patella.

To facilitate region-based analysis of cartilage thickness, the knee MR image was divided into 14 structural ROIs, including anterior (A), central (C), and posterior (P) regions of the medial (M)/lateral (L) femur (F), tibia (T), and patella (P), with exemplar delineation in Figure 2. The above regions were defined in accordance with WORMS (17).

### Neural Network With Automatic Segmentation

A neural network was used to segment knee joint from MR images automatically. Similar to Norman et al. (11), we chose the two-dimensional U-Net (20) architecture, a widely used method in medical image segmentation, to segment six regions (i.e., femur, tibia, patella, and corresponding cartilages) slice-to-slice. Considering our limited training data size, weight decay and data-augmentation techniques such as slice-wise spatial transformation of rotation, translation, and rescaling were applied to prevent overfitting. Also, subject-wise histogram-matching was adopted for better generalization. The neural network was implemented in PyTorch and trained on an Nvidia GeForce GTX 1080 Ti Graphics Card.

In this study, the Dice similarity coefficient (DSC) was used to evaluate the accuracy of segmentation. DSC was calculated with *DSC*(*A, B*) = 2|*A*∩*B*|/(|*A*|+|*B*|), where *A* and *B* were the reference segmentation (i.e., manual labeling) and automatic segmentation, respectively, whereas the operator |·| counts the number of elements in the set. The value of DSC ranges from 0 to 1, and the higher value indicates better segmentation performance. We used 27 subjects (570 slices) for training and 20 subjects (471 slices) for testing. The segmentation DSCs of femoral cartilage, tibial cartilage, and patellar cartilage were 0.87 ± 0.01, 0.82 ± 0.01, and 0.76 ± 0.04, respectively, which were comparable with manual labeling (see Table 2). The reproducibility experiment results of the segmentation model were shown in the Supplementary Materials (see Supplementary Figure 1.)

**Table 2:** Results of automatic and manual segmentation accuracy of knee joint in DSC (calculated based on individuals).

**ROI**	**DSC of automatic segmentation**	**DSC of manual segmentation**
Femur	0.9727 ± 0.0066	0.9671 ± 0.0056
Femoral cartilage	0.8660 ± 0.0140	0.7769 ± 0.0215
Tibia	0.9651 ± 0.0191	0.9583 ± 0.0091
Tibial cartilage	0.8237 ± 0.0219	0.7248 ± 0.0335
Patella	0.9471 ± 0.0169	0.9306 ± 0.0121
Patellar cartilage	0.7581 ± 0.0398	0.7156 ± 0.0462

*ROI, region of interest; DSC, dice similarity coefficient*.

### Cartilage Thickness Quantification

With the development and degeneration of normal human cartilage, the cartilage thickness at different regions of the knee joint changes gradually. On the basis of the automatic segmentation results described earlier, we could calculate the cartilage thickness (21) following the steps later. First, we used the femur as an example and generated the signed distance maps of femur and femur cartilage. The value in the distance map records the distance from the specific location to the nearest boundary point, with negative/positive signs for locations inside/outside the segmented region of femur or femur cartilage. Third, we calculated the first-order gradient maps from the corresponding signed distance maps. Fourth, by performing a dot-product operation on the two gradient maps, we determined inner cartilage boundary points (facing toward femur bone) if dot-products were negative or outer cartilage boundary points (away from femur) if positive. Finally, we used the outer boundary points and computed the distance map for the femur cartilage, from which the distances could be read for all inner boundary points.

Next, we used an atlas with predefined ROIs to complete the segmentation of individual regions per subject. The atlas was labeled to delineate all 14 ROIs. By deforming the atlas to align with each subject image via one-by-one registration, we acquired ROIs for all collected images. Then, for each subject MR image, we calculated the cartilage thickness and derived the average measure for each region (11). The cartilage thickness measures, corresponding to the 14 delineated ROIs, allowed us to compare across different age/sex groups and track the longitudinal thinning pattern accordingly. Error analysis of cartilage morphology and quality assessment of the quantitative analysis of the cartilage thickness was shown in the supplementary materials (see Supplementary Table 1, Supplementary Figures 2–15).

### Statistical Analyses

Cartilage thickness of different regions, ages, sexes, and sides were compared using ANOVA pairwise comparisons method Tukey. Tukey's method is used in ANOVA to create confidence intervals for all pairwise differences between factor level means while controlling the family error rate to a level specified. An alpha level of 0.05 was set for statistical significance, and all tests were two-tailed. In the Tukey simultaneous test results, the 95% simultaneous confidence level implies that all the confidence intervals contain the true differences. If an interval does not contain zero, the corresponding means are significantly different.

Regression analysis of cartilage thickness changes in 14 regions with sex/laterality was performed with a fit regression model. We created a categorical condition variable, i.e., sex/laterality. Then, we included the interaction term for input × condition, i.e., age × sex/laterality. We fit the regression model with input (continuous independent variable), condition (main effect), and input × condition (interaction effect). The coefficient of age × sex/laterality represents the difference between the coefficient for males and females. The *p*-value lower than 0.05 indicates that this difference is statistically significant. In other words, we could conclude that sex/laterality affects the relationship between input and output. The consistent (or inconsistent) change with age implies no significant (or significant) difference between sexes/lateralities. The analyses mentioned earlier were performed in Minitab Version 19.1, and the figures were drawn in Python 3.7.

## Results

### Comparisons of Cartilage Thickness Across 14 Regions of Interest

**Figure 3:**
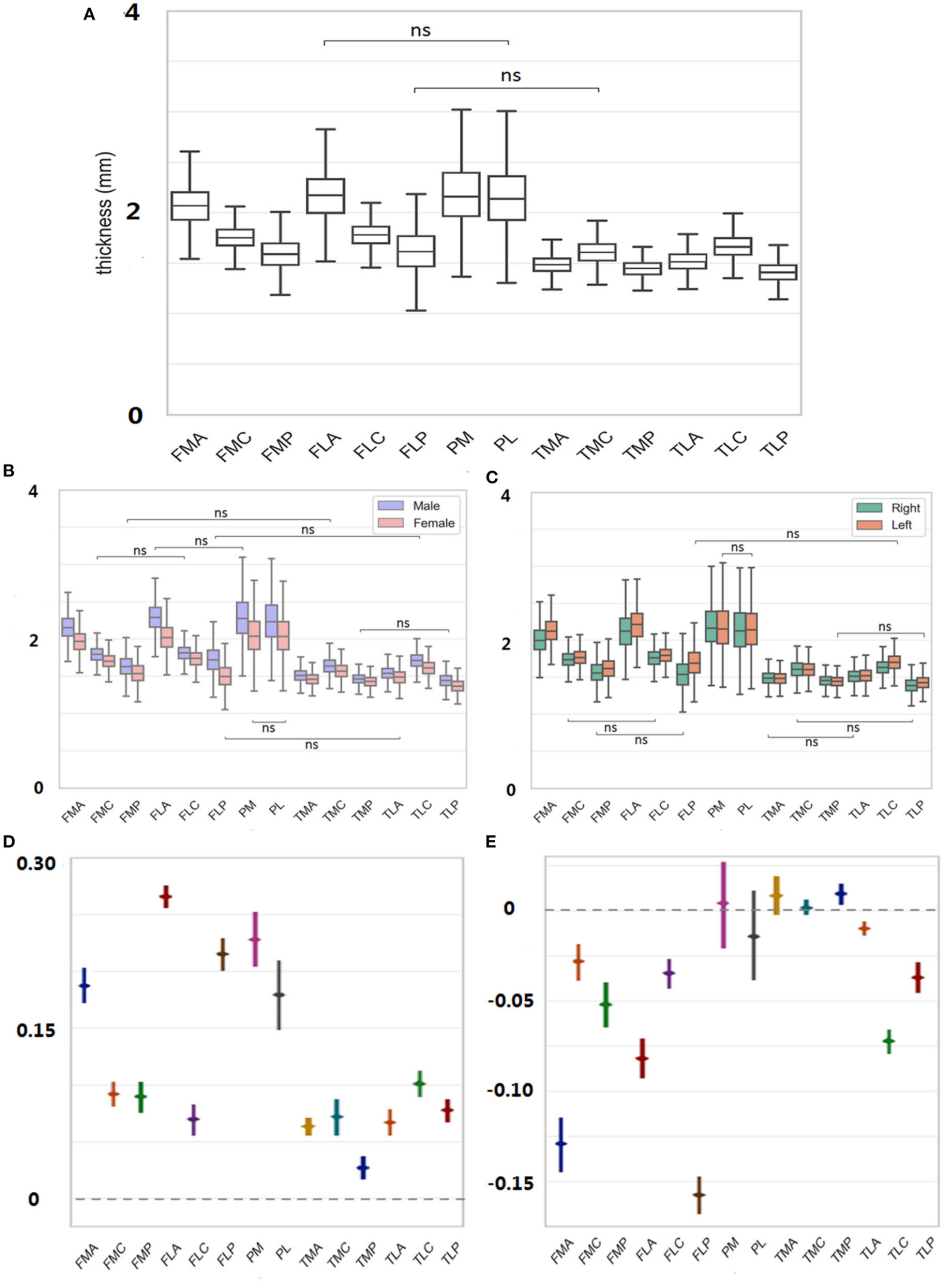
**(A)** Thickness of each ROI averaged was measured over all subjects. **(B)** For each ROI, thickness was further decomposed to male/female sex. **(C)** Thickness was further decomposed to left/right side. **(D)** Male to female difference in the cartilage thickness reading was provided for each region. **(E)** Right to left difference in cartilage thickness reading was provided for each region. ns = *p* > 0.05.

The average thickness of each ROI over all subjects was plotted in Figure 3A. The comparisons among all 14 ROIs revealed prevalent thickness differences in the knee joint. Particularly, PM owned the highest cartilage thickness (2.18 ± 0.32 mm; for convenience, the “mm” unit would be skipped in the following thickness reading). When comparing corresponding A/C/P regions between femur and tibia (i.e., FMA vs. TMA, FMC vs. TMC, etc.), femur always led in the reading {FMA vs. TMA: 2.06 ± 0.20 vs. 1.48 ± 0.10 [difference of means 0.58 ± 0.01, 95% confidence interval (CI) 0.56–0.59], FMC vs. TMC: 1.75 ± 0.12 vs. 1.60 ± 0.12 (0.15 ± 0.01, 0.13–0.16); *p* < 0.05 in all above tests}. In the femur particularly, the cartilage thickness in the non-load-bearing areas (i.e., FMA and FLA) was greater than the load-bearing C areas (FMC and FLC) and P areas (FMP and FLP) (FMA vs. FMC/FMP: 2.06 ± 0.20 vs. 1.75 ± 0.12/1.59 ± 0.16, FLA vs. FLC/FLP: 2.15 ± 0.24 vs. 1.77 ± 0.13/1.61 ± 0.22, *p* < 0.05 in all tests mentioned earlier). On the contrary, for the tibia, the C areas of TMC and TLC led in the thickness measuring, compared with A and P areas (TMC vs. TMA/TMP: 1.60 ± 0.12 vs. 1.48 ± 0.10 mm/1.44 ± 0.08, TLC vs. TLA/TLP:1.66 ± 0.13 vs. 1.51 ± 0.11/1.40 ± 0.11; *p* < 0.05 in tests mentioned earlier).

For each ROI, the thickness reading was further separated into male and female sexes and was compared in Figures 3B,D. The findings were consistent with those mentioned earlier without considering the sex factor. For both females and males, the cartilage thickness in the non-load-bearing areas was greater than the load-bearing C and P areas for the femur. Meanwhile, across the upper and lower corresponding A/C/P regions, the thickness in the femur was always greater than in the tibia. When referring to the sex difference, the cartilage thickness tended to be always thicker in men than in women (FMA, men vs. women, 2.16 ± 0.18 vs. 1.97 ± 0.16, FMC, 1.80 ± 0.11 vs. 1.70 ± 0.11, 0.10 ± 0.01; FMP, 1.63 ± 0.16 vs. 1.54 ± 0.15, 0.09 ± 0.01, *p* < 0.05 in all tests mentioned earlier). The largest margin for the sex-related cartilage thickness occurred in FLA (0.27 ± 0.01), contrary to the smallest difference in TMP (0.04 ± 0.01).

The thickness measured in Figure 3A was also separated into the left and right sides, and the result for the laterality is shown in Figures 3C,E. The cartilage thickness was thicker in left knees than in right knees, which was statistically significant in all femoral regions (FMA, left vs. right, 2.13 ± 0.18 vs. 2.00 ± 0.20; FMC, 1.77 ± 0.12 vs. 1.73 ± 0.12, *p* < 0.05 in tests mentioned earlier). In all TL regions, the same findings were revealed (TLA, left vs. right, 1.52 ± 0.11 vs. 1.50 ± 0.11; TLC, 1.70 ± 0.14 vs. 1.62 ± 0.12; TLP, 1.43 ± 0.10 vs. 1.38 ± 0.11; *p* < 0.05 in tests mentioned earlier). However, no significant difference between the left and right sides was detected in TMA, TMC, and the patella (*p* > 0.05).

### Cartilage Thickness Changes With Ages

**Figure 4:**
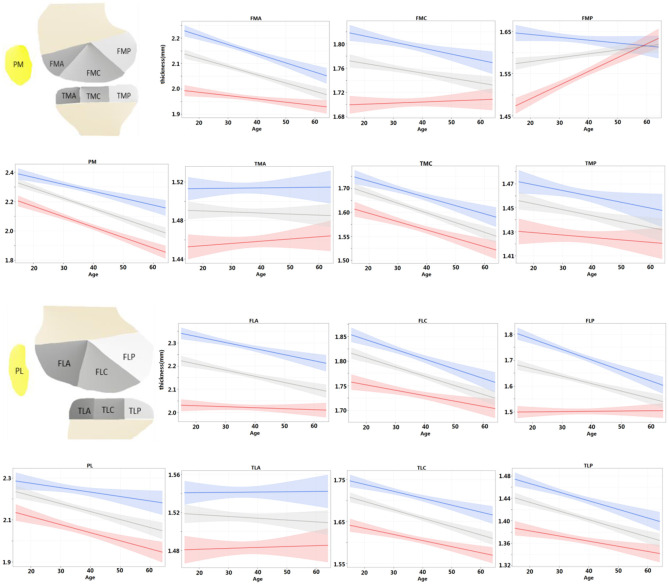
For each ROI, regression model of cartilage thickness with respect to ages was derived for both men and women and compared with population at same time. Shading represented 95% CIs.

For each ROI, the regression model of the cartilage thickness with respect to ages was derived for both men and women. The models for all 14 ROIs are shown in Figure 4. The tendency of cartilage thickness change with aging was consistent (i.e., no significant difference between females and males) from all tibial ROIs and PL, yet inconsistent (i.e., a significant difference between females and males) in PM and femoral ROIs except for FLC. Among the eight consistent regions, the cartilage thickness in the TMA and TLA regions did not change significantly with age (age coefficient > 0, *p* > 0.05), whereas others became thinner. Meanwhile, considering the inconsistent six regions, the cartilage thickness in FMP increased slightly alongside age for women (age coefficient > 0, *p* < 0.05) and in other ROIs decreased temporally (age coefficient < 0, *p* < 0.05). The thinning speed in the FMA area was faster in men than in women (*p* < 0.05 for all age × sex coefficients) and, in contrast, slower for men in the PM area (*p* < 0.05 for all age × sex coefficients). Additionally, the cartilage degenerated in the ROIs of FMC, FLA, and FLP for men (age coefficient <0, *p* < 0.05), whereas women's changes were not statistically significant (age coefficient <0, *p* > 0.05).

**Figure 5:**
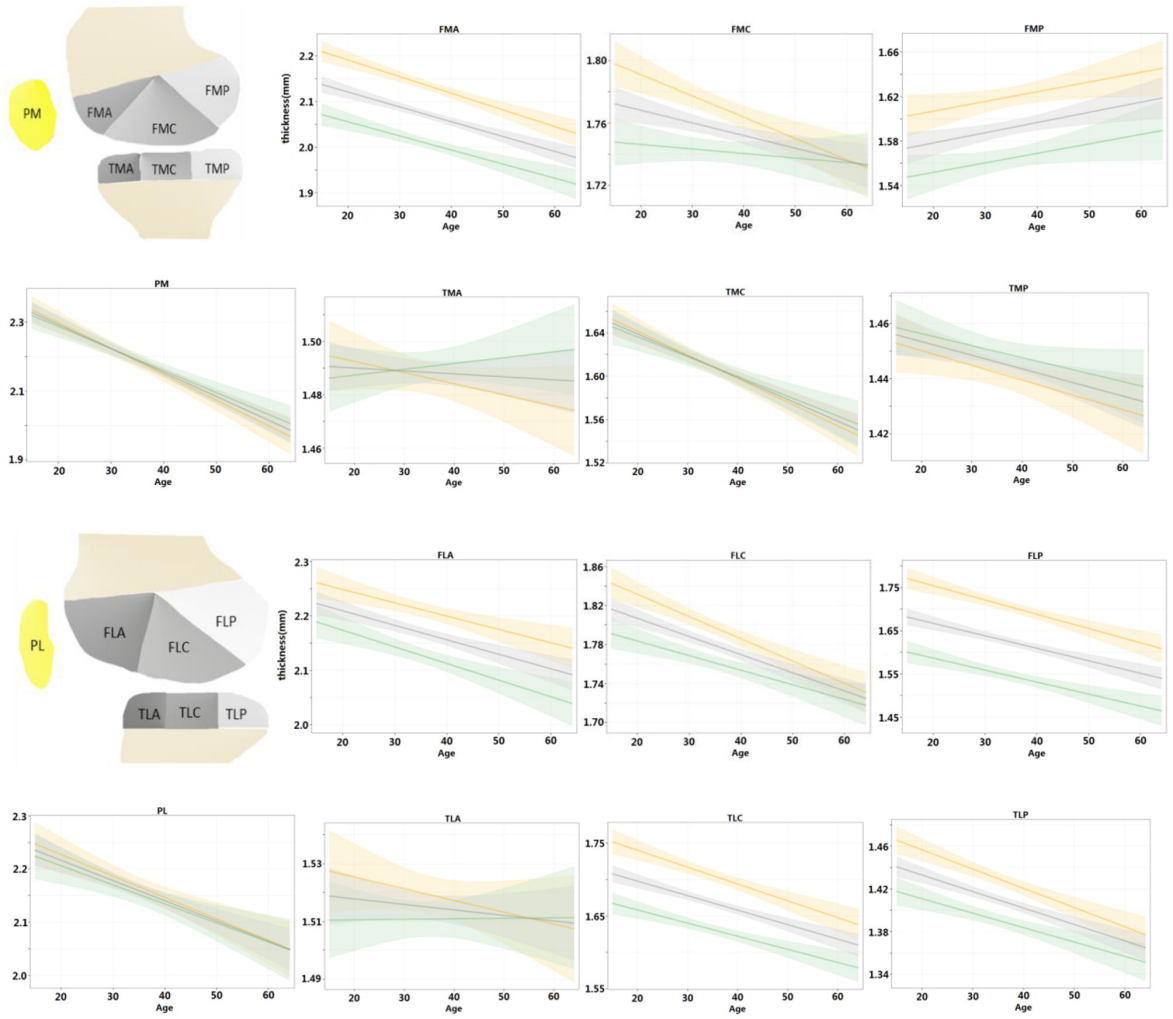
Regression models were fitted for left and right knees, respectively, and compared with the population at the same time. Shading represented 95% CIs.

Further, the regression models were fitted for the left and right knees and compared with the entire population in Figure 5. For the left and right knees, the temporal changes were consistent in all areas except for FMC (*p* > 0.05 for all age × laterality coefficients except for FMC). Among the consistent regions, the cartilage thickness in the TMA and TLA regions did not change significantly with age (age coefficient <0, *p* > 0.05), FMP increased with age (age coefficient > 0, *p* < 0.05), and others degenerated (age coefficient <0, *p* < 0.05). That is, both knees tended to degenerate in a synchronized manner.

## Discussion

We used the deep learning method to automatically segment the cartilage and calculated the cartilage thickness in 14 individual regions of the whole knee joint. For the first time, we specified the characteristics of cartilage thickness across 14 nonoverlapping ROIs of the knee joint and tracked their thinning alongside aging, which provided a standardized reference to reveal the pathogenesis of knee OA potentially. From our findings, male cartilage thickness was greater than female in all age groups across the knee joint. Furthermore, the thinning was inconsistent from PM and all femoral ROIs except for FLC between men and women. Age and sex do play a role in cartilage thinning despite the diversity in men and women. Differences in cartilage thickness may further suggest varying risks of developing knee OA between women and men (22, 23).

So far, only one study using deep learning to assess the thickness of femoral cartilage has been reported (24). Particularly, the segmentation model produced DSCs between 0.77 and 0.88 in the femoral cartilage compartments of several landmarks. Our model was applied to the whole knee joint in this work and produced comparable DSCs between 0.76 and 0.87 for the femoral, tibial, and patellar cartilage compartments. Moreover, compared with quantifying the cartilage thickness near the landmarks in medial and lateral femur only (24), we generalized to the whole knee joint, including femur, tibia, and patella. We made a comprehensive quantitative analysis of cartilage thickness in 14 regions according to WORMS (17), which was widely accepted for the accurate positioning of clinical assessment of cartilage thinning or defects. In addition, we further established a model of cartilage thickness variation alongside ages through robust statistical methods. Our findings further validated the previous work (24) upon the Chinese population and concluded that age and sex significantly impacted cartilage thickness.

Our study filled the vacancy with increasing ages in a relatively younger population (mean age: 36 years), comparing our results with another large population-based cross-sectional study, i.e., Framingham OA study investigating knee MR imaging in the middle-aged and elderly population (mean age: 62 years) (25–27). The results indicated that the medial posterior femoral region was more vulnerable in the whole knee joint. Specifically, the Framingham OA study (27) found that MR-detected cartilage damages were highly prevalent in the medial posterior tibiofemoral joints. Our findings, on the other hand, showed that the cartilage thickness in the non-load-bearing FMA area was greater than the load-bearing FMC and FMP areas in radiographically normal knees, which was possibly attributed to the factor that FMC and FMP had a higher chance of natural cartilage wear. Unfortunately, the thickness of the 14 individual regions considering body mass index information was not reported and needed further study. In addition, we found the cartilage thickness in the TMA and TLA areas did not change significantly with aging. It may be because these two regions do not have much contact or association with other areas during joint movement.

Based on the regression analysis of men and women, we found that the cartilage thickness in the FMP region of bilateral knees of women increased with age. Previous studies (28, 29) also reported that the cartilage thickness in the FMP area could increase in OA knees compared with non-OA knees without considering the sex differences. Therefore, we infer that the thickening of cartilage in the FMP area is a physiological change related to hormones. However, the biological mechanism underlying such a response is still unclear in the literature. Post-traumatic spontaneous reparation (30) and the measurement error should also be taken into account. We look forward to more studies in the future related to the histo-biological processes involved in this thickening area.

Given the regression results of left and right knees, we found that the cartilage thinning pattern was always consistent except for FMC only. Moreover, previous studies (31, 32) also reported that knees from subjects with bilaterally radiographically normal knees had lower cartilage thinning scores. It implies that the cartilage thinning is generally synchronized in the left and right knees. However, more information about the habitual use of limbs needs to be collected to get a more accurate interpretation.

There existed several limitations in our study. Firstly, our sample size was skewed to a certain extent, resulting in the relatively young age distribution (median age: 34 years; mean age: 36 years). Secondly, our segmentation protocol did not include high-resolution three-dimensional imaging, and we would further use the three-dimensional sequences of the knee joint to improve our segmentation model in the following research. Thirdly, because the assessment was blindly performed by two practicing board-certified musculoskeletal radiologists without identification and health information of patients, we did not assess more information such as body mass index and medical history such as common chronic diseases. Finally, we did not accomplish follow-up exams in this cohort to obtain and compare its cartilage information to make more accurate predictions, which would be a potential direction for future research.

In conclusion, prevalent cartilage thickness differences existed across individual regions in the knee joint, by considering the factors of age, sex, and side, with respect to a large population of 2,481 subjects and their 3-T MR images. Cartilage thinning alongside ages was identified for both men and women. The thinning pattern was consistent in the tibia while partially inconsistent from patellar and femoral cartilages between women and men. The findings earlier may provide references to detect cartilage anomaly.

## Data Availability Statement

The original contributions presented in the study are included in the article/Supplementary Materials, further inquiries can be directed to the corresponding author/s.

## Ethics Statement

The studies involving human participants were reviewed and approved by Ethics Committee of Shanghai Sixth People's Hospital. Written informed consent to participate in this study was provided by the participants' legal guardian/next of kin.

## Author Contributions

KX and QW: computer programming and manuscript editing. WY and HZ: guarantors of integrity of entire study and study concepts/study design. LS and KX: statistical analysis and experimental studies. LS and JH: data acquisition or data analysis/interpretation. LS, JZ, YX, JG, and YH: literature research and manuscript revision. All authors contributed to the article and approved the submitted version.

## Conflict of Interest

The authors declare that the research was conducted in the absence of any commercial or financial relationships that could be construed as a potential conflict of interest.

## Abbreviations

ANOVA: analysis of variance
CI: confidence interval
F: female
FMA: medial anterior femur
FMC: medial central femur
FMP: medial posterior femur
FLA: lateral anterior femur
FLC: lateral central femur
FLP: lateral posterior femur
L: left
M: male
OA: osteoarthritis
PFJ: patellofemoral joint
PM: medial patella
PL: lateral patella
R: right
ROI: region of interest
TFJ: tibiofemoral joint TMA, medial anterior tibia
TMC: medial central tibia
TMP: medial posterior tibia
TLA: lateral anterior tibia
TLC: lateral central tibia
TLP: lateral posterior tibia
WORMS: Whole-Organ Magnetic Resonance Imaging Score.
