# Supplementary material for: Knee Cartilage Thickness Differs Alongside Ages: A 3-T Magnetic Resonance Research Upon 2,481 Subjects via Deep Learning
Source: Front Med (Lausanne). 2021 Feb 9;7:600049. doi: 10.3389/fmed.2020.600049 (PMC7900571; doi:10.3389/fmed.2020.600049)
Supplement: Supplementary file 1 [file Table_1.docx]

Knee cartilage thickness differs alongside ages:

A 3T MR research upon 2481 subjects via deep learning

Supplementary materials

**Abbreviations:**

FMA=medial anterior femur, FMC=medial central femur, FMP=medial posterior femur, FLA=lateral anterior femur, FLC=lateral central femur, FLP=lateral posterior femur, PM=medial patella, PL=lateral patella, TMA=medial anterior tibia, TMC=medial central tibia, TMP=medial posterior tibia, TLA=lateral anterior tibia, TLC=lateral central tibia, TLP=lateral posterior tibia, FC = femoral cartilage, LTC = lateral tibial cartilage, MTC = medial tibial cartilage, PC = patellar cartilage.

1. **Reproducibility of the segmentation model**

We selected an independent training set and redone the segmentation and showed that both training sets provided equivalent and precise results (**Figure S1**). In femur, the dice coefficient of model 1 vs. model 2 was 0.97±0.01 vs. 0.98±0.00; in femoral cartilage, 0.87±0.01 vs. 0.88±0.02; in tibia, 0.97±0.02 vs. 0.97±0.01; in tibial cartilage, 0.82±0.02 vs. 0.81±0.02; in patella, 0.95±0.02 vs. 0.96±0.01; in patellar cartilage, 0.76±0.04 vs. 0.77±0.04.


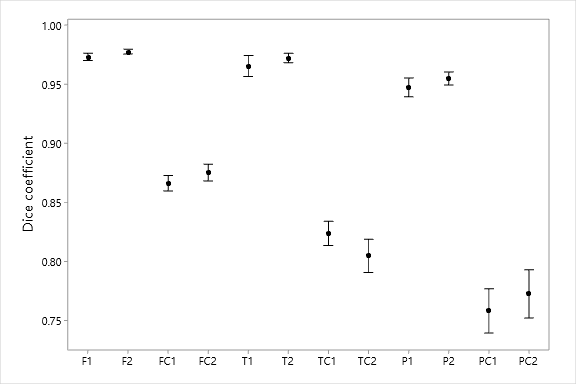


**Figure S1.** After the segmentation model was trained with independent training sets, the model had good repeatability.

Note: F1 = femur in training set 1, F2 = femur in training set 2, FC1 = femoral cartilage in training set 1, FC2 = femoral cartilage in training set 2, T1 = tibia in training set 1, T2 = tibia in training set 2, TC1 = tibial cartilage in training set 1, TC2 = tibial cartilage in training set 2, P1 = patella in training set 1, P2 = patella in training set 2, PC1 = patellar cartilage in training set 1, PC2 = patellar cartilage in training set 2.

training set 1 = 27, testing set = 20

training set 2 = 27, testing set = 20

1. **Error analysis of Morphology**

According to our scanning parameters, the spacing is 0.303 x 0.303 x 3.3mm, and the maximum error of our MAE is about one pixel (see **Table S1**), which is acceptable. In addition, compared with the segmentation result errors of other study^1^ (mainly compared with MAE), when scanning parameters with a resolution of 0.346 x 0.346 x 0.7 mm, the maximum MAE is about 0.4315, which is an acceptable range. We compared the difference between the two methods, i.e. ground truth and automatic segmentation by using Wilcoxon signed-rank test at the α<0.0001 level.

**Table S1.** MAE, MSE and RMSE for cartilage thickness based on manual (true) and automatic (prediction) segmentation of 14 regions

| ROI | MSE(mm^2^) | RMSE(mm) | CoV(RMS) (%) | MAE(mm) | *p*-value |
| --- | --- | --- | --- | --- | --- |
| FMA | 0.11556 | 0.33994 | 15.213 | 0.30768 | <0.0001 |
| FMC | 0.06824 | 0.26124 | 14.083 | 0.23829 | <0.0001 |
| FMP | 0.36454 | 0.60377 | 31.721 | 0.58832 | <0.0001 |
| FLA | 0.04725 | 0.21737 | 9.518 | 0.18567 | <0.0001 |
| FLC | 0.07410 | 0.27221 | 14.453 | 0.24836 | <0.0001 |
| FLP | 0.28382 | 0.53274 | 28.203 | 0.50884 | <0.0001 |
| TMA | 0.03499 | 0.18704 | 12.033 | 0.14713 | <0.0001 |
| TMC | 0.06256 | 0.25012 | 14.667 | 0.19972 | <0.0001 |
| TMP | 0.01588 | 0.12602 | 8.755 | 0.10546 | <0.0001 |
| TLA | 0.03071 | 0.17526 | 12.943 | 0.14559 | <0.0001 |
| TLC | 0.07388 | 0.27181 | 11.188 | 0.20849 | <0.0001 |
| TLP | 0.03687 | 0.19201 | 15.446 | 0.16100 | <0.0001 |
| PM | 0.17224 | 0.41501 | 17.399 | 0.32388 | <0.0001 |
| PL | 0.19699 | 0.44384 | 18.504 | 0.35963 | <0.0001 |

| ROI | MSE(mm^2^) | RMSE(mm) | CoV(RMS) (%) | MAE(mm) | *p*-value |
| --- | --- | --- | --- | --- | --- |
| FC | 0.15892 | 0.39865 | 19.851 | 0.34619 | <0.0001 |
| LTC | 0.04716 | 0.21715 | 13.544 | 0.17169 | <0.0001 |
| MTC | 0.03781 | 0.19444 | 12.413 | 0.15077 | <0.0001 |
| PC | 0.18462 | 0.42967 | 17.963 | 0.34176 | <0.0001 |

Note: MAE = mean absolute error, MSE = mean square error, RMSE = root mean square error，CoV (RMS) = root mean square (RMS) coefficient of variation (CoV).

1. **Quality assessment of the quantitative analysis of the cartilage thickness**

We performed the Bland-Altman plots showing the automated evaluation of cartilage thickness vs the manual derived cartilage thicknesses of the 14 provided regions. The Bland-Altman plots (**Figure S2 – Figure S15**) showed the bias and limits of agreement. As for the testing data analysis, it showed comparable precision in manual and automatic procedures in the quantitative analysis of the cartilage.


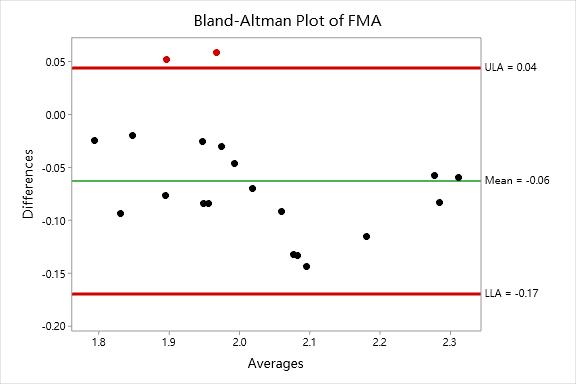


**Figure S1.** Bland-Altman plots show comparison of thickness calculations in the region of FMA produced from manual and automatic segmentation methods. Estimation mean of the differences was -0.06 and estimation of standard deviation of the differences was 0.05.


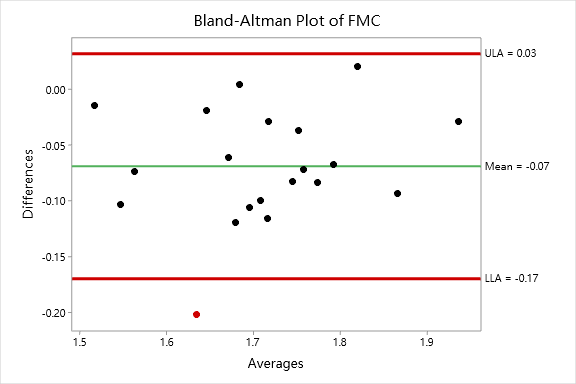


**Figure S2.** Bland-Altman plots show comparison of thickness calculations in the region of FMC produced from manual and automatic segmentation methods. Estimation mean of the differences was -0.07 and estimation of standard deviation of the differences was 0.05.


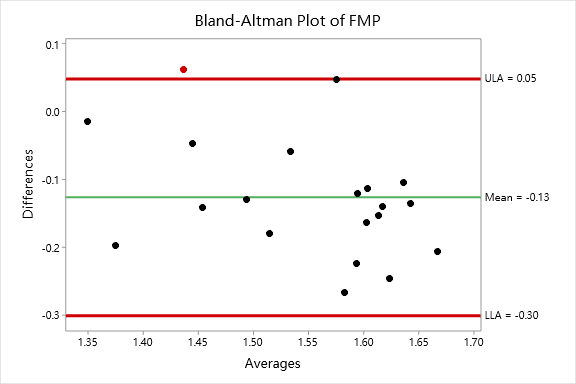


**Figure S3.** Bland-Altman plots show comparison of thickness calculations in the region of FMP produced from manual and automatic segmentation methods. Estimation mean of the differences was - -0.13 and estimation of standard deviation of the differences was 0.09.


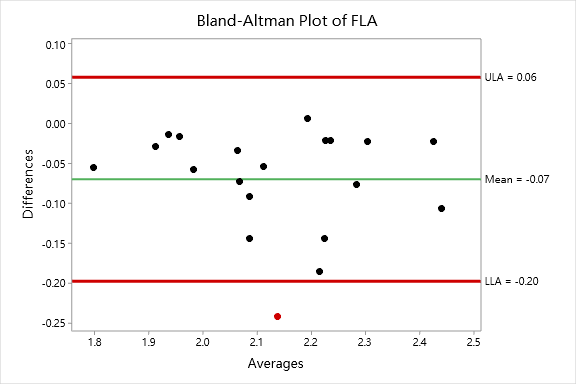


**Figure S4.** Bland-Altman plots show comparison of thickness calculations in the region of FLA produced from manual and automatic segmentation methods. Estimation mean of the differences was -0.07 and estimation of standard deviation of the differences was 0.07.


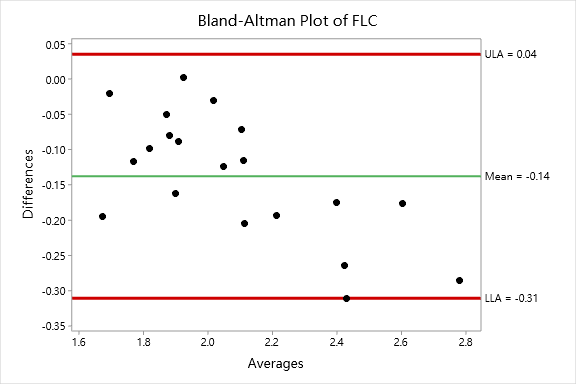


**Figure S5.** Bland-Altman plots show comparison of thickness calculations in the region of FLC produced from manual and automatic segmentation methods. Estimation mean of the differences was -0.14 and estimation of standard deviation of the differences was 0.09.


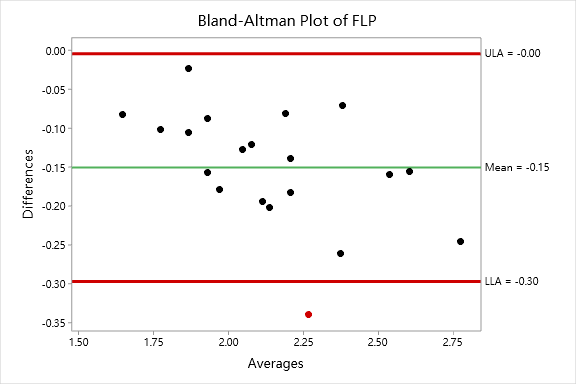


**Figure S6.** Bland-Altman plots show comparison of thickness calculations in the region of FLP produced from manual and automatic segmentation methods. Estimation mean of the differences was -0.15 and estimation of standard deviation of the differences was 0.07.


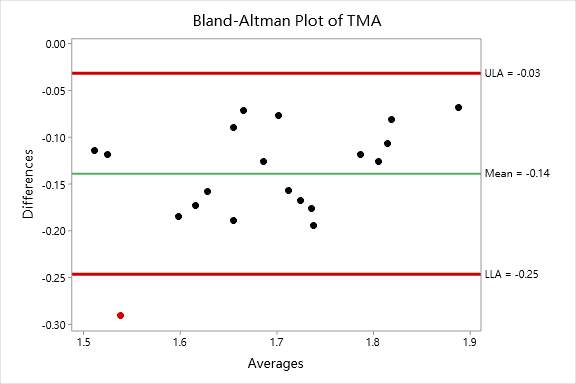


**Figure S7.** Bland-Altman plots show comparison of thickness calculations in the region of TMA produced from manual and automatic segmentation methods. Estimation mean of the differences was -0.14 and estimation of standard deviation of the differences was 0.05.


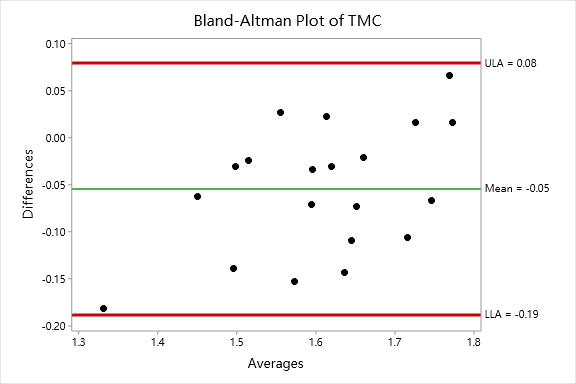


**Figure S8.** Bland-Altman plots show comparison of thickness calculations in the region of TMC produced from manual and automatic segmentation methods. Estimation mean of the differences was -0.05 and estimation of standard deviation of the differences was 0.07.


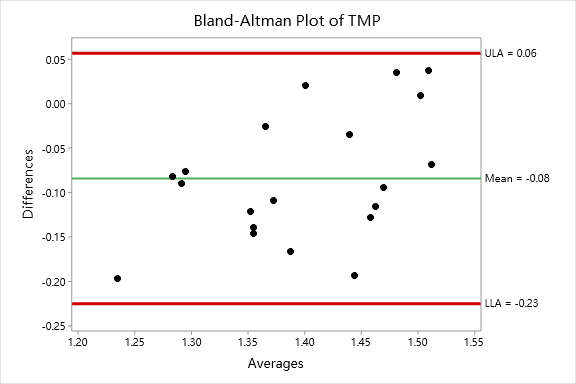


**Figure S9.** Bland-Altman plots show comparison of thickness calculations in the region of TMP produced from manual and automatic segmentation methods. Estimation mean of the differences was -0.08 and estimation of standard deviation of the differences was 0.07.


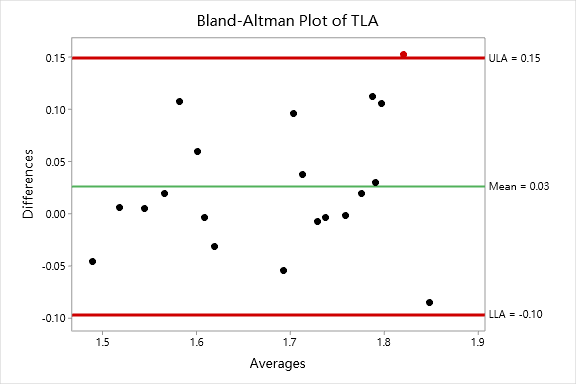


**Figure S10.** Bland-Altman plots show comparison of thickness calculations in the region of TLA produced from manual and automatic segmentation methods. Estimation mean of the differences was 0.03 and estimation of standard deviation of the differences was 0.06.


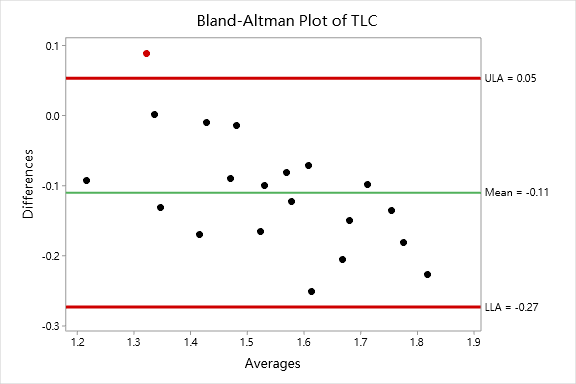


**Figure S11.** Bland-Altman plots show comparison of thickness calculations in the region of TLC produced from manual and automatic segmentation methods. Estimation mean of the differences was -0.11 and estimation of standard deviation of the differences was 0.08.


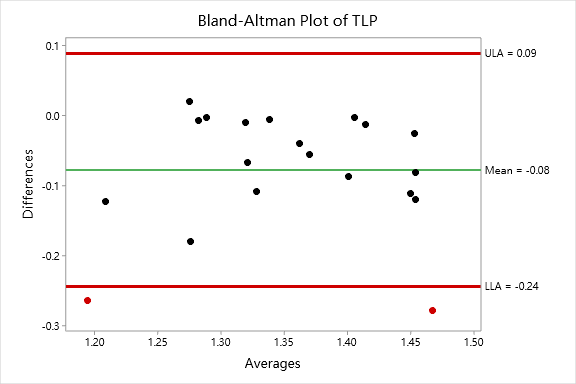


**Figure S12.** Bland-Altman plots show comparison of thickness calculations in the region of TLP produced from manual and automatic segmentation methods. Estimation mean of the differences was -0.08 and estimation of standard deviation of the differences was 0.09.


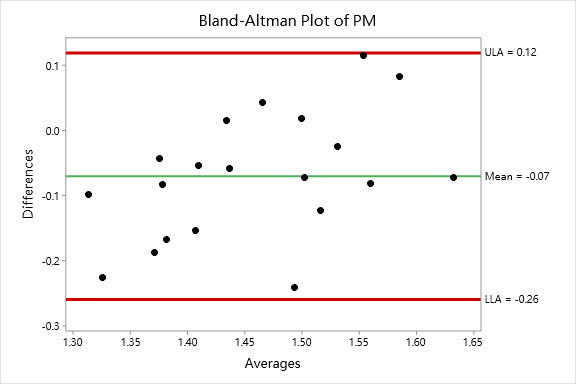


**Figure S13.** Bland-Altman plots show comparison of thickness calculations in the region of PM produced from manual and automatic segmentation methods. Estimation mean of the differences was -0.07 and estimation of standard deviation of the differences was 0.10.


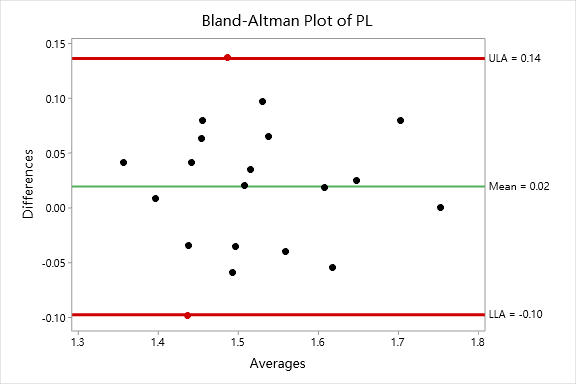


**Figure S14.** Bland-Altman plots show comparison of thickness calculations in the region of PL produced from manual and automatic segmentation methods. Estimation mean of the differences was 0.02 and estimation of standard deviation of the differences was 0.06.

1. **Results of regression equations (Table S2-S4)**

**Table S2. Results of regression equations for all subjects.** An alpha level of 0.05 was set for statistical significance and all tests were two-tailed. *P* value of age coefficient represents the statistical significance of the changing tendency of cartilage thickness.

| **Regression Equation** | **age Coef** | **P** |
| --- | --- | --- |
| FMA=2.1858 - 0.003254 age | -0.003254 | * |
| FMC=1.78419 - 0.000810 age | -0.00081 | * |
| FMP=1.5600 + 0.000918 age | 0.000918 | * |
| FLA=2.2625 - 0.002660 age | -0.00266 | * |
| FLC=1.84403 - 0.001861 age | -0.001861 | * |
| FLP=1.7250 - 0.002891 age | -0.002891 | * |
| PM=2.4315 - 0.006944 age | -0.006944 | * |
| PL=2.2922 - 0.003802 age | -0.003802 | * |
| TMA=1.49223 - 0.000110 age | -0.00011 | ns |
| TMC=1.67900 - 0.002006 age | -0.002006 | * |
| TMP=1.46330 - 0.000495 age | -0.000495 | * |
| TLA=1.52160 - 0.000191 age | -0.000191 | ns |
| TLC=1.73735 - 0.001986 age | -0.001986 | * |
| TLP=1.46387 - 0.001543 age | -0.001543 | * |

**Table S3. Results of regression equations for females and males.** An alpha level of 0.05 was set for statistical significance and all tests were two-tailed. *P* value of age coefficient represents the statistical significance of the changing tendency of cartilage thickness, and of age*gender coefficient represents the statistical significance of the difference in rate of change between women and men.

| **ROI** | **Gender** | **Regression Equation** | **age Coef** | **P** | **age*gender Coef** | **P** |
| --- | --- | --- | --- | --- | --- | --- |
| FMA | F | FMA=2.0127 - 0.001300 age | -0.0013 | * | -0.002307 | * |
|  | M | FMA=2.2828 - 0.003608 age | -0.003608 | * |  |  |
| FMC | F | FMC=1.6968 + 0.000183 age | 0.000183 | ns | -0.001185 | * |
|  | M | FMC=1.8333 - 0.001002 age | -0.001002 | * |  |  |
| FMP | F | FMP=1.4253 + 0.003250 age | 0.00325 | * | -0.003928 | * |
|  | M | FMP=1.6563 - 0.000677 age | -0.000677 | ns |  |  |
| FLA | F | FLA=2.0379 - 0.000431 age | -0.000431 | ns | -0.002192 | * |
|  | M | FLA=2.3805 - 0.002623 age | -0.002623 | * |  |  |
| FLC | F | FLC=1.7737 - 0.001094 age | -0.001094 | * | -0.00087 | ns |
|  | M | FLC=1.8828 - 0.001964 age | -0.001964 | * |  |  |
| FLP | F | FLP=1.4978 + 0.000112 age | 0.000112 | ns | -0.004181 | * |
|  | M | FLP=1.8636 - 0.004068 age | -0.004068 | * |  |  |
| PM | F | PM=2.3113 - 0.007111 age | -0.007111 | * | 0.00239 | * |
|  | M | PM=2.4597 - 0.004719 age | -0.004719 | * |  |  |
| PL | F | PL=2.1934 - 0.003852 age | -0.003852 | * | 0.00174 | ns |
|  | M | PL=2.3177 - 0.002116 age | -0.002116 | * |  |  |
| TMA | F | TMA=1.4493 + 0.000231 age | 0.000231 | ns | -0.000197 | ns |
|  | M | TMA=1.51260 + 0.000034 age | 0.000034 | ns |  |  |
| TMC | F | TMC=1.6324 - 0.001729 age | -0.001729 | * | 0.000033 | ns |
|  | M | TMC=1.6987 - 0.001696 age | -0.001696 | * |  |  |
| TMP | F | TMP=1.43339 - 0.000202 age | -0.000202 | ns | -0.000284 | ns |
|  | M | TMP-1.47893 - 0.000486 age | -0.000486 | * |  |  |
| TLA | F | TLA=1.4795 + 0.000095 age | 0.000095 | ns | -0.000066 | ns |
|  | M | TLA=1.5403 + 0.000029 age | 0.000029 | ns |  |  |
| TLC | F | TLC=1.6628 - 0.001448 age | -0.001448 | * | -0.0002 | ns |
|  | M | TLC=1.7713 - 0.001648 age | -0.001648 | * |  |  |
| TLP | F | TLP=1.4005 - 0.000916 age | - 0.000916 | * | -0.000616 | ns |
|  | M | TLP=1.49710 - 0.001532 age | - 0.001532 | * |  |  |

**Table S4. Results of regression equations for left and right knees.** An alpha level of 0.05 was set for statistical significance and all tests were two-tailed. *P* value of age coefficient represents the statistical significance of the changing tendency of cartilage thickness, and of age*laterality coefficient represents the statistical significance of the difference in rate of change between left and right knees.

| **ROI** | **Laterality** | **Regression Equation** | **age Coef** | **P** | **age*laterality Coef** | **P** |
| --- | --- | --- | --- | --- | --- | --- |
| FMA | L | FMA=2.2641-0.003651 age | -0.003651 | * | -0.000557 | ns |
|  | R | FMA=2.1174-0.003094 age | -0.003094 | * |  |  |
| FMC | L | FMC=1.8181-0.001363 age | -0.001363 | * | -0.001073 | * |
|  | R | FMC=1.7519-0.000290 age | -0.00029 | ns |  |  |
| FMP | L | FMP=1.5894+0.000874 age | 0.000874 | * | 0.000021 | ns |
|  | R | FMP=1.5349+0.000853 age | 0.000853 | ns |  |  |
| FLA | L | FLA=2.2974-0.002445 age | -0.002445 | * | 0.000606 | ns |
|  | R | FLA=2.2346-0.003051 age | -0.003051 | * |  |  |
| FLC | L | FLC=1.8771 - 0.002282 age | -0.002282 | * | 0.000789 | ns |
|  | R | FLC=1.8134 - 0.001494 age | -0.001494 | * |  |  |
| FLP | L | FLP=1.8204 - 0.003306 age | -0.003306 | * | 0.000528 | ns |
|  | R | FLP=1.6421 - 0.002777 age | -0.002777 | * |  |  |
| PM | L | PM=2.4475 - 0.007475 age | -0.007475 | * | 0.0011 | ns |
|  | R | PM=2.4145 - 0.006379 age | -0.006379 | * |  |  |
| PL | L | PL=2.3077 - 0.004035 age | -0.004035 | * | 0.00045 | ns |
|  | R | PL=2.2777 - 0.003589 age | -0.003589 | * |  |  |
| TMA | L | TMA=1.5008 - 0.000416 age | -0.000416 | ns | 0.000635 | ns |
|  | R | TMA=1.4830 + 0.000218 age | 0.000218 | ns |  |  |
| TMC | L | TMC=1.6850 - 0.002178 age | -0.002178 | * | 0.000354 | ns |
|  | R | TMC=1.6728 - 0.001825 age | -0.001825 | * |  |  |
| TMP | L | TMP=1.46092 - 0.000538 age | -0.000538 | * | 0.000103 | ns |
|  | R | TMP=1.46501 - 0.000436 age | -0.000436 | ns |  |  |
| TLA | L | TLA=1.5335 - 0.000407 age | -0.000407 | ns | 0.000424 | ns |
|  | R | TLA=1.5102 + 0.000017 age | 0.000017 | ns |  |  |
| TLC | L | TLC=1.7861 - 0.002308 age | -0.002308 | * | 0.000511 | ns |
|  | R | TLC=1.6941 - 0.001798 age | -0.001798 | * |  |  |
| TLP | L | TLP=1.4927 - 0.001803 age | -0.001803 | * | 0.000454 | ns |
|  | R | TLP=1.4378 - 0.001350 age | -0.00135 | * |  |  |

**Reference**

1. Norman B, Pedoia V, Majumdar S. Use of 2D U-Net Convolutional Neural Networks for Automated Cartilage and Meniscus Segmentation of Knee MR Imaging Data to Determine Relaxometry and Morphometry. Radiology. 2018 Jul;288(1):177-185.
